# Functional role of WW domain‐containing proteins in tumor biology and diseases: Insight into the role in ubiquitin‐proteasome system

**DOI:** 10.1096/fba.2019-00060

**Published:** 2020-02-21

**Authors:** Shenq‐Shyang Huang, Li‐Jin Hsu, Nan‐Shan Chang

**Affiliations:** ^1^ Graduate Program of Biotechnology in Medicine Institute of Molecular and Cellular Biology National Tsing Hua University Hsinchu Taiwan, ROC; ^2^ Department of Medical Laboratory Science and Biotechnology National Cheng Kung University College of Medicine Tainan Taiwan, ROC; ^3^ Institute of Molecular Medicine National Cheng Kung University College of Medicine Tainan Taiwan, ROC; ^4^ Department of Neurochemistry New York State Institute for Basic Research in Developmental Disabilities Staten Island NY USA; ^5^ Graduate Institute of Biomedical Sciences College of Medicine China Medical University Taichung Taiwan, ROC

**Keywords:** degradation, neural diseases, proteasome, tumorigenesis, ubiquitination, WW domain

## Abstract

The ubiquitin‐proteasome system (UPS) governs the protein degradation process and balances proteostasis and cellular homeostasis. It is a well‐controlled mechanism, in which removal of the damaged or excessive proteins is essential in driving signal pathways for cell survival or death. Accumulation of damaged proteins and failure in removal may contribute to disease initiation such as in cancers and neurodegenerative diseases. In this notion, specific protein‐protein interaction is essential for the recognition of targeted proteins in UPS. WW domain plays an indispensable role in the protein‐protein interactions during signaling. Among the 51 WW domain‐containing proteins in the human proteomics, near one‐quarter of them are involved in the UPS, suggesting that WW domains are crucial modules for driving the protein‐protein binding and subsequent ubiquitination and degradation. In this review, we detail a broad spectrum of WW domains in protein‐protein recognition, signal transduction, and relevance to diseases. New perspectives in dissecting the molecular interactions are provided.

**Abbreviations**
ACK1: activated Cdc42‐associated kinase 1
AMOTL1: angiomotin like 1
BH3: Bcl‐2 homology 3
BMP: bone morphogenetic proteins
Cx43: Connexin 43
Cyld: CYLD Lysine 63 Deubiquitinase
DMBA: 7,12‐dimethylbenz(a)anthracene
EGF: epidermal growth factor
EGFR: epidermal growth factor receptor
EGR2: early growth response 2
ENaC: epithelial sodium channel
GDNF/Ret: glial cell‐derived neurotrophic factor
HECT: homologous to the E6‐AP carboxyl terminus
Htt: huntingtin
Id1: inhibitor of DNA binding 1
IRF‐3: interferon regulatory factor‐3
ITCH: itchy E3 ubiquitin protein ligase
JNK2: c‐jun N‐terminal kinase 2
KLF5: Krüppel‐like factor 5
LATS1: large tumor suppressor 1
LPS: lipopolysaccharides
MAPK: mitogen‐activated protein kinase
MEKK2: mitogen‐activated protein kinase kinase kinase 2
NDRG1: *N*‐myc Downstream Regulated 1
NEDD4: the neural precursor cell expressed, developmentally downregulated protein 4
NEDD4L: neural precursor cell expressed, developmentally downregulated 4‐like
NEDL1/HECW1: HECT, C2, and WW domain‐containing E3 ubiquitin protein ligase 1
NEDL2/HECW2: HECT, C2, and WW domain‐containing E3 ubiquitin protein ligase 2
NF‐kB: nuclear factor kappa light chain enhancer of activated B cells
PCP: planar cell polarity
PI3K: phosphoinositide 3‐kinase
Pk1: Prickle 1
PTEN: phosphatase and tensin homolog
RASSF5: ras association domain family member 5
RBR: Ring‐Between‐Ring
RTKs: receptor tyrosine kinases
SH3: Src homology domain 3
SHH: sonic Hedgehog
SMURF1: Smad ubiquitination regulatory factor 1
SMURF2: Smad ubiquitination regulatory factor 2
SOD1: superoxide dismutase‐1
TACE: tumor necrosis factor‐converting enzyme
TAK1: transforming growth factor beta‐activated kinase 1
TGF‐β: transforming growth factor beta
TLR: toll‐like receptor
TRAP‐δ: translocon‐associated protein‐δ
TRIF: TIR‐domain‐containing adapter‐inducing interferon‐β
UPS: ubiquitin‐proteasome system
WWP1: WW domain‐containing E3 ubiquitin protein ligase 1
WWP2: WW domain‐containing E3 ubiquitin protein ligase 1
YY1: Yin Yang 1

## 1 INTRODUCTION

Eukaryotic cells possess at least two proteolytic systems to tackle misfolded or damaged proteins. One is the ubiquitin‐proteasome system (UPS), which is target‐specific and is responsible for most of the proteolytic events. The other one is autophagy, which accounts for approximately 10%‐20% of the cellular proteolysis.^1^ The term “ubiquitination” is coined to describe the covalent conjugation of ubiquitin molecules to the targeted protein for degradation via the proteasomal system. The central mechanism of protein ubiquitination involves three steps.^2^ First, the ubiquitin‐activating enzyme E1 activates ubiquitin through the formation of thiol ester with the carboxyl group of Gly76 of ubiquitin. Second, the resulting activated *C*‐terminus of ubiquitin is transferred to the ubiquitin‐conjugating E2 enzyme. Lastly, the E3 enzyme recognizes the substrate and catalyzes the transfer of ubiquitin from the E2 to the substrate. To date, there are at least 600 E3 ligases for determining the fate of tens of thousands of proteins in the human body.^3^ At least four distinct groups of E3 ligases have been classified, including HECT‐type, RING‐finger‐type, U‐box domain proteins, and RBR E3s.^3^, ^4^, ^5^

In particular, among the E3s, HECT‐type ubiquitin E3 ligases have the *C*‐terminal HECT domain and the *N*‐terminal extension, which contains a distinct domain recognizing specific target proteins. There are 51 WW domain‐containing proteins identified in the human proteome. Among them, nine WW domain‐containing proteins are involved in UPS, and these proteins belong to the NEDD4 subfamily of HECT‐type ubiquitin E3 ligase. In other words, all the E3 ligases in the NEDD4 subfamily contain WW domains in their tertiary structures, implying a unique role of the WW domain in determining the substrate specificity and protein‐protein interactions during signaling. Disruption of the normal signaling process might lead to enhanced disease development and progression.

## 2 WW DOMAIN‐CONTAINING PROTEINS IN THE PROTEIN UBIQUITINATION‐PROTEASOME SYSTEM

WW domain is a compact protein module comprising 35‐40 amino acids and two conserved tryptophan (W) residues spaced apart by ~20 amino acids.^6^, ^7^, ^8^, ^9^, ^10^ Since the discovery of WW domains in 1994, more than 2000 WW domain‐containing proteins have been identified. Similar to the SH3 domain, WW domain binds the proline‐rich region in proteins. However, the consensus binding sequence for WW domain is different from that of SH3 domain.^11^, ^12^ This property confers the unique binding feature of WW domain‐containing proteins with their targets. WW domains are divided into four groups.^9^ The group I WW domain recognizes the core consensus PPxY or LPxY motif (PY motif); group II recognizes PPLP motifs (PL motif); group III recognizes proline‐rich sequences with arginine residues ([R]‐R/K/x‐PP or PP‐R/x‐[R]; PR motifs); group IV recognizes phospho(serine/threonine)‐proline containing sequences (p(S/T)P motif). In addition, a newly defined group V has a substrate specificity of the uninterrupted polyproline sequences (p/f)PPPPP.^8^

WW domain‐containing proteins not only play roles in signal transduction, but also participate in regulating protein post‐translational modifications. Certain WW domain‐containing proteins are involved in the UPS. Interestingly, all the WW domain‐containing proteins participate in the UPS belonging to the NEDD4 ubiquitin ligase subfamily,^13^ suggesting the unique role of the WW domain in regulating protein ubiquitination. Proteins in NEDD4 ubiquitin ligase subfamily have similar features, including an *N*‐terminal C2 domain, two to four WW domains, and a *C*‐terminal HECT domain.^13^ The C2 domain is responsible for membrane recruitment upon Ca^2+^ stimulation,^14^ and the HECT domain catalyzes the transfer of the ubiquitin thioester intermediater from E3 ubiquitin ligase to the substrate.^15^ Therefore, the NEDD4 ubiquitin ligase subfamily is categorized into the HECT‐type ubiquitin ligase. Among them, nine proteins containing WW domains include NEDD4, NEDD4L, ITCH, WWP1, WWP2, SMURF1, SMURF2, NEDL1/HECW1, and NEDL2/HECW2.^13^ Almost all the WW domains in NEDD4 family E3 ligases recognize the PY motif, while phosphoylation of the serine or threonine residue preceding the PY motif, that is, p(S/T)P, leads to physically interact with the group IV WW domains.

## 3 ROLE OF WW DOMAIN‐CONTAINING E3 LIGASE IN CANCERS AND MANY DISEASES

Failure of protein degradation is the common cause of accumulation of the malfunctioned and/or oncogenic proteins. These aberrant protein aggregates may contribute to tumorigenesis, neuronal diseases. and other devastating disorders.^16^ While the WW domains in the HECT‐type ubiquitin ligases are involved in protein‐protein recognition and physical binding, a dispensable role of the WW domain in the protein ubiquitination/proteasomal degradation in the particular signaling event is suggested.

The majority of the tumor cells are originated from the epithelial lineages.^17^, ^18^ Under harsh environmental conditions, rapid cell replacement to cover the damaged or worn out tissues is necessary to maintain the physical barrier that protects tissues from pathogenic invasion and chemical erosion. In such a circumstance, the signals that dictate those cellular events must be well controlled. Aberrant or uncontrolled signaling frequently causes excessive cell growth and contributes to tumor development.

### 3.1 EGFR family proteins and regulation by WW domain proteins

The EGFR family of receptor tyrosine kinases (RTKs) plays an important role in epithelial cell homeostasis. The EGFR family includes EGFR/ErbB1/HER1, ErbB2/HER2, ErbB3/HER3, and ErbB4/HER4. Each protein has a distinct role in controlling cellular function. Abnormal EGFR signaling and EGFR amplification cause tumorigenesis and malignancy.^19^, ^20^ The WW domain‐containing E3 ligases participate in EGFR signaling and regulate the downstream signaling, including NEDD4, NEDD4L, WWP1, and ITCH. Notably, ErbB4/HER4 is a common target for those E3 ligases (Table 1).

**Table 1:** Representative WW domain proteins and their binding targets

Protein	Tandemly repeated WW domains^a^	Binding proteins	Target sequences/motifs	Reference
**NEDD4** **Diseases:** Prostate and breast cancers^23^, ^24^ Liddle syndrome^101^, ^102^ Parkinson's disease^106^ **Signaling:** Signaling by ERBB4^21^, ^22^	#1 to 4	HER3	972‐PPRY‐975	23
	#2 & 3	ErbB4/HER4‐Cyt1	1032‐PPIY‐1035 1053‐PPAY‐1056 1298‐PPPY‐1301	21, 22
	#1 to 3 (#2 is more specific to phosphor‐PY motif)	Connexin Cx43	279‐pSPMpSPPGY‐286	98, 99
	WW domains (not specified)	RTP801	Proline rich region close to the N‐terminus	106
	WW domains (not specified)	Numb	Proline rich region between a.a.199‐415 and a.a.416‐593	78
	#2 to 4	ENaC β subunit	615‐PPNY‐618	103
**NEDD4L** **Disease:** Prostate and gastric cancers^48^, ^49^ Liddle syndrome^105^ **Signaling:** TGF‐beta and receptor signaling activates SMADs^47^, ^142^ Voltage‐gated Sodium channels (Na_v_)^108^ Wnt signaling^50^	# 2 to 4	ENaC β subunit	615‐PPNY‐618	102, 105
	# 2 & 3	ACK1	632‐PPAY‐ 635	25
	#2 & 3 (#2 is stronger)	Smad2	220‐TPPPGY‐225	47
	#2 & 3 (#2 is stronger)	Smad3	179‐TPPPGY‐184	47
	#2 & 3 (#2 is stronger)	Smad7	207‐PPPPY‐211	142
	#3 (stronger than 2)	Dvl1	546‐PPCFPPAYQDPG‐557	50
	#3 (stronger than 2)	Dvl2	561‐YSPQPPPYHELS‐572	50
	#3 (stronger than 2)	Dvl3	655‐PPGVPPLYGPPM‐666	50
	#4	Na_v_ (Na_v_1.1‐ Na_v_1.8)	PPSY in the *C*‐terminal tail	108
**ITCH** **Disease:** Autoimmune disease, multi‐system, with facial dysmorphism and organomegaly^53^, ^143^ Soft tissue sarcoma, ovarian, and breast cancers^54^, ^55^ **Signaling:** ERBB4 Signaling^21^ Hippo signaling^56^, ^57^ Ras signaling^61^	#3 & 4	P73	479‐SHCTPPPPYHA‐489	69
	WW domains (not specified)	P63	501‐PPPY‐504 in TAp63α; 446‐PPPY‐449 in ΔNp63α	68
	WW domains (not specified)	Cyld	482‐PPFY‐485	93
	WW domains (not specified)	LATS1	556‐PPPY‐559	58
	WW domains (not specified)	RASSF5A	10‐RPYP‐14	61
	WW domains (not specified	Ptch1	PPPY in the C‐terminal domain and PPXY motif in the central loop	123
	#1 & 4	ErbB4/HER4‐Cyt1	PPPIY; PPPAY only in Cyt1; PPPPY	21
**WWOX** **Diseases:** Spinocerebellar ataxia, autosomal recessive epileptic encephalopathy, early infantile^144^, ^145^ **Signaling:** WWOX and transcriptional regulation with many transcriptional factors^146^, ^147^ Hyal‐2/ WWOX/Smad4 signaling^148^ WWOX/p53 signaling^149^ Apoptosis, autophagy, and bubbling cell death^150^	#1	ITCH	661‐LPFY‐664; 877‐LPPY‐880	62
**WWP1** **Disease:** Spastic Paraplegia 20, Autosomal Recessive^151^ **Signaling:** ERBB4 signaling^31^, ^33^ Cellular senescence^89^	WW domains (not specified)	KLF5	324‐PPPSY‐328	83
	WW domains (not specified)	P53	Proline‐rich region in a.a.68‐91	70
	#1 & 3	ErbB4‐CYT1	PPPAY in CYT‐1	33
	#1 to 4	HER4	PY2 PY3	31
	#1 to 4 (#1 is the strongest)	P63	501‐PPPY‐504 in TAp63α; 446‐PPPY‐449 in ΔNp63α	68
**WWP2** Plantar Fasciitis^152^ **Signaling:** SMAD Signaling Network^153^ PTEN signaling^79^ Notch signaling^90^ T cell and TLR signaling^91^^,^ 92	#1 & 2 (#2 stronger)	Oct4	Proline‐rich motif (not specified)	154
	#1 & 4	EGR2	165‐PPPPPPPPY‐173; 204‐PPPSY‐208	91
	N.A. (Not addressed)	PTEN	phosphatase domain a.a. 100‐187	79
	#2 to 4	TRIF	N‐terminal and TIR domain	92
	#1 to 4	Notch3	2208‐PPPY‐2211 in the PEST domain	90
**HECW1** **Diseases:** Neuroblastoma^72^ Familial amyotrophic lateral sclerosis^72^, ^114^ **Signaling:** Wnt signaling^72^ JNK/c‐Jun signaling^72^	#1 & 2	Dvl1	545‐PPPCFPPAY‐553; 642‐PPPHP‐646; 657‐PPGGPP‐662	72
	Linker between C2 and WW domains	Mutant SOD1	N. A. (not specified)	72
	#1 & 2	translocon‐associated protein‐δ (TRAP‐δ)	N. A. (not specified)	72
	C2, Linker, WW domain (without HECT)	P53	Proline‐rich motif	73
**HECW2** Hirschsprung's disease^117^, ^118^ **Signaling:** p73 transcriptional activity^115^ Tight junction/endothelium stability^116^	#1 & 2	p73α and p73β	405‐PPSY‐408 and 484‐PPPY‐487	115
	#1 & 2	AMOLT1	310‐PPEY‐313; 367‐PPEY‐370	116
**SMURF1** **Diseases:** Wolfram Syndrome^155^ Cerebral cavernous malformations^156^, ^157^ Important for brain development^122^ **Signaling**: Smad signaling^34^, ^36^, ^47^, ^52^, ^142^ Sonic hedgehog signaling pathway^122^	#1 & 2	MEKK2	166‐PPGY‐169	38
	#1 & 2	Smad1	222‐TPPPAY‐227	47,52
	#1 & 2	Smad5	219‐TPPPAY‐224	47,52
	WW domains (not specified)	Smad6	linker domain (181‐331) SPPPPYSR	36
	WW2	Smad7	207‐PPPPY‐211	34, 142
	WW domains (not specified)	Ptch1	643‐PPPY‐646; 1313‐PPPY‐1316	122
	WW domains (not specified)	Runx2	418‐YHTYLPPPYPGSSQ‐431	37
**SMURF2** **Diseases:** Important for brain development^122^ **Signaling**: Signaling by BMP^39^, ^40^, ^41^, ^142^ Sonic hedgehog signaling pathway^122^	#2 & 3	Smad2 (244‐434)	PY motif, not specified	39
	#2 & 3	Smad2	220‐pT‐PPPGY‐225	40
	#2 & 3	Smad3	179‐pT‐PPPGY‐184	40
	WW3	Smad7	207‐PPPPY‐211	41,142
	WW domains (not specified)	KLF5	312‐TPPPSY‐317	88
	WW domains (not specified)	Id1	N‐terminal of ID1 (1‐65) (No PY motif)	89
	WW domains (not specified)	YY1	PPDY	75
	WW domains (not specified)	Ptch1	PY motif, not specify the a.a. position	122

a Tandemly repeated WW domains, including #1 = WW domain 1 or WW1; #2 = WW domain 2 or WW2; #3 = WW domain 3 or WW3; #4 = WW domain 4 or WW4.

NEDD4 binds ErbB4/HER4 through its second and third WW domains.^21^ Also, NEDD4 physically binds the PY motifs in the Cyt1 region of ErbB4/HER4.^22^ Mutations in PY motifs impair the binding interactions, and thereby lead to a reduced ubiquitination level and increased protein stability of ErbB4/HER4.

**Figure 1:**
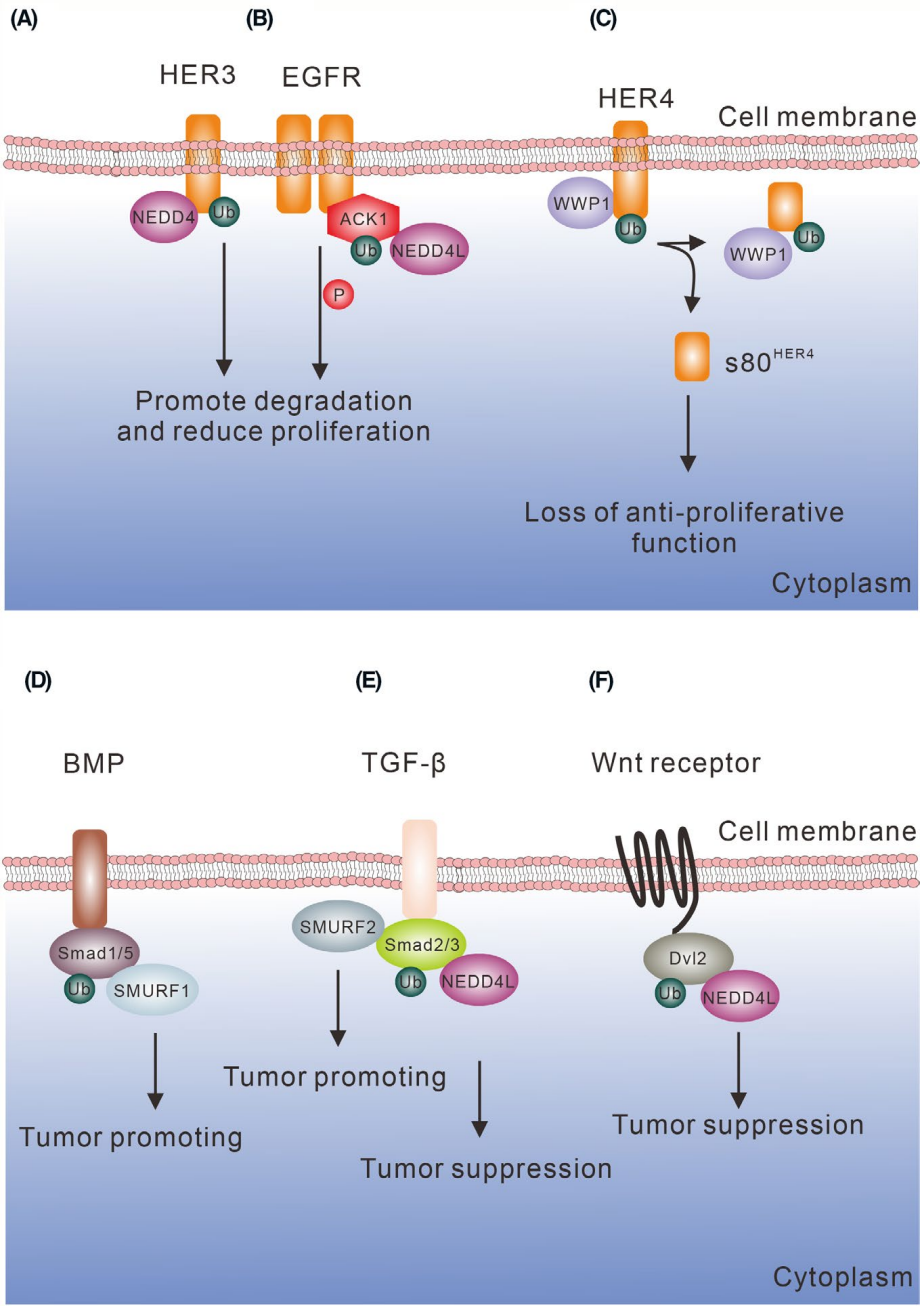
EGFR family proteins and regulation by WW domain proteins. (A) NEDD4 ubiquitinates ErbB3/HER3 and ErbB4/HER4 and reduces the survival signaling, while loss of NEDD4 promotes cell proliferation. (B) ACK1 is a downstream adaptor of EGFR signaling. NEDD4L ubiquitinates ACK1 and promotes ACK1 degradation and reduces cell proliferation. (C) WWP1 ubiquitinates the cytoplasmic region of ErbB4/HER4 and blocks the anti‐proliferative function of ErbB4/HER4. (D‐F) SMURF1, SMURF2, and NEDD4L participate in the regulation of BMP, TGF‐β, and Wnt signaling pathways, respectively. Both SMURF1 and SMURF2 regulate Smads. NEDD4L ubiquitinates Dvl2 and reduces Wnt signaling. All the aforementioned ubiquitinated proteins are subjected to degradation

Similarly, the WW domains of NEDD4 physically bind the *C*‐terminal PY motif in ErbB3/HER3 for ubiquitination and degradation (Figure 1A). Loss of NEDD4 increases the kinase activities of ErbB3/HER3 and downstream proteins such as PI3K and MAPK for promoting cell proliferation. Moreover, NEDD4 and ErbB3/HER3 protein expression levels are inversely correlated in prostate cancer tissues, suggesting that loss of NEDD4 promotes malignant prostatic cell growth.^23^ In contrast, inhibition of the nonreceptor tyrosine kinase PKY2 induces ErbB3/HER3 degradation and increases NDRG1 expression in breast cancer cells. NDRG1 promotes the interaction of ErbB3/HER3 and NEDD4 and enhances ErbB3/HER3 ubiquitination and degradation. As a result, combined targeting of both EGFR and PKY2 reduces cancer cell survival and promotes tumor regression in triple‐negative breast tumors.^24^

NEDD4L has a structure similar to NEDD4. NEDD4L does not directly mediate EGFR ubiquitination but regulates EGFR signaling through an EGFR‐associated protein, ACK1. ACK1 is a tyrosine kinase mediating the downstream signaling of EGFR. ACK1 interacts with EGFR and blocks EGFR from ubiquitination.^25^ Mechanistically, the second and third WW domains of NEDD4L bind to the PY motif in ACK1, and the binding promotes ACK1 ubiquitination and degradation upon EGF stimulation (Figure 1B). Hence, a previous study reported that NEDD4L does not directly regulate EGFR, and the participation of other E3 ubiquitin ligases might be required.^26^

ErbB4/HER4 has at least four isoforms originated from alternative splicing. Upon activation, ErbB4/HER4 is cleaved by TACE, resulting in the generation of an 80‐kDa membrane‐associated ErbB4/HER4 (m80). Only the isoforms containing the JM‐a domain can be recognized and cleaved by TACE. The m80 is further cleaved by γ‐secretase to produce a soluble 80‐kDa fragment, s80^HER4^. The cleavage of Cyt1 and Cyt2 gives rise to a distinct s80^HER4^ that has different biological functions in the nucleus.^27^, ^28^, ^29^ Naresh et al showed that s80^HER4^ derived from JM‐a Cyt1 has a proapoptotic BH3 domain and promotes apoptotic cell death.^30^ Also, nuclear s80^HER4^ increases the expression of WWP1, which specifically binds the three PY motifs in JM‐a Cyt1 and promotes the degradation of the membrane‐bound full‐length and the 80‐kDa membrane‐associated forms, but not the s80^HER4^. Notably, loss of the C2 domain in WWP1 promotes s80^HER4^ degradation, suggesting that the unique spatial distribution of WWP1 is a key factor for regulating the ErbB4/HER4 protein level.^31^ Clinically, ErbB4/HER4 is a well‐recognized prognosis marker.^32^ WWP1 promotes ErbB4/HER4 degradation, thereby blocking the anti‐proliferation and differentiation functions of ErbB4/HER4 (Figure 1C). Other E3 ligases also participate in the regulation of ErbB4/HER4. For example, ITCH and HECW1 reduce the ErbB4/HER4 protein level in breast cancer.^21^, ^33^ However, the detailed molecular mechanism of how HECW1 regulates ErbB4/HER4 has not been addressed.

Taken together, WW domain‐containing E3 ubiquitin ligases play an indispensable role in controlling EGFR signaling and maintaining the balance between proliferation and anti‐proliferation function of EGFR. Loss of WW domain‐containing E3 ubiquitin ligases contributes to the imbalanced epithelial proliferation and tumorigenesis.

### 3.2 SMURF1/2 and NEDD4L control BMP and TGF‐β pathways

SMURF1 and NEDD4L are involved in the BMP and TGF‐β pathways. SMURF1 recognizes the phospho‐PY motif in Smad1/5 that are phosphorylated sequentially by CDK8/9 and GSK3 (Figure 1D). Similarly to Smad1/5, Smad7 is another target of SMURF1. SMURF1 binds the nuclear Smad7 and promotes the ubiquitination and degradation of TGF‐β type I receptor.^34^ Notably, SMURF1 is required for Smad7 to undergo cytoplasmic translocation and membrane recruitment to the receptor,^35^ implying the indispensable role of SMURF1 in the TGF‐β receptor signaling.

SMURF1 has an anti‐inflammatory function. SMURF1 binds Smad6 and promotes MyD88 polyubiquitination at K231 and K262 for proteasomal degradation. Degradation of MyD88 terminates the upstream LPS‐induced inflammatory signaling and TLR4 activation and inflammatory signaling.^36^ Runx2 is one of the transcription factors involved in bone development and chondrocyte maturation. SMURF1 has been shown to downregulate the Runx2 protein level. SMURF1 binds the *C*‐terminal PY motif of Runx2. However, this binding interaction is not sufficient for driving Runx2 degradation. It has been determined that Smad6 is required for SMURF1‐mediated Ruxn2 degradation,^37^ suggesting a novel role of Smad6 in promoting SMURF1 function. Mice lacking SMURF1 exhibit increased bone mass and osteoblast activity. SMURF1 suppresses bone morphogenesis and formation by modulating the MEKK2 and JNK2 activities.^38^ Specifically, this regulation is made by the interaction between the WW domains in SMURF1 and the PY motif in MEKK2. Moreover, the phosphor‐PY motif is a putative substrate for SMURF1 to turn off MEKK2 signaling and reduce the sensitivity of osteoblasts to TGFβ/BMP stimulation.^38^

SMURF1 and SMURF2 share a similar protein structure. SMURF1 has two WW domains, and SMURF2 has three. SMURF2 physically binds Smad1, Smad2, and Smad3, and has the highest binding affinity to Smad2. SMURF2 targets Smad2 for ubiquitination and degradation upon TGF‐β stimulation (Figure 1E).^39^ Compelling evidence from Tang's study showed that upon type I TGF‐β receptor activation, Smad2 and Smad3 are phosphorylated at T220 and T179 preceding the PY motif, respectively.^40^ The phosphorylation of Smad2/3 transforms the sequence from group I WW domain‐targeted sequences to group IV and promotes SMURF2 binding. SMURF2 does not downregulate Smad2 and Smad3 protein levels. However, SMURF2 binds Smad3 and induces Smad3 monoubiquitination for inhibiting the complex formation of Smad3‐Smad3 and Smad3‐Smad4. Hence, monoubiquitination mediated by SMURF2 reduces the nuclear translocation of Smad proteins and the expression of TGF‐β response genes.^40^ Similar to SMURF1, SMURF2 also binds and induces the degradation of Smad7 and TGF‐β receptors.^41^

TGF‐β signaling exerts cancer suppression or promotion depending on the cancer stage. How cancer cell switches from the growth inhibition of TGF‐β toward growth promotion is largely unknown. In the early stage, TGF‐β signaling is associated with favorable prognosis; while in the late stage, it correlates with poor prognosis, as the cancer cells metastasize to target organ sites.^42^ Loss of TGF‐β signaling has been shown in breast, prostate, and renal cell carcinoma.^43^, ^44^, ^45^ As mentioned above, SMURF2 restricts the TGF‐β signaling via monoubiquitination for blocking nuclear translocation of Smad complexes, as well as undergoing proteasomal degradation of Smads and TGF‐β receptors. Expression of SMURF2 and Smad2 proteins inversely correlates in many tumor tissues. Patients with higher levels of SMURF2 expression in tumors have shorter progression‐free survival.^46^

In addition to SMURF1/2, NEDD4L recognizes the phospho‐PY motif in Smad2/3^47^ (Figure 1E). In prostate and gastric cancers, deficient expression of NEDD4L contributes to a higher tumor grade and poor prognosis,^48^, ^49^ suggesting that NEDD4L boosts the TGF‐β/Smad signaling to suppress tumorigenesis. Furthermore, the third WW domain of NEDD4L is associated with Dvl2 downregulation (Figure 1F). Binding of NEDD4L with Dvl2 leads to suppression of Wnt/Dvl2 signaling and inhibition of colorectal cancer growth.^50^ NEDD4L downregulation is frequently found in tumors, and this correlates with tumor progression.^51^ Thus, control of protein degradation is important for regulating the cellular response to stimuli, especially for TGF‐β/Smads. Both SMURF1/2 and NEDD4L cooperate with each other to regulate the activation/degradation of Smads in BMP and TGF‐β signal pathways, again suggesting the importance in the control of biological outcome.^52^

In summary, WW domain‐containing E3 ubiquitin ligases such as SMURF1/2 and NEDD4L participate in the regulation of BMP, TGF‐β, and Wnt signaling and control the cellular response to the external stimuli such as TGF‐β and Wnt ligands.

### 3.3 Multi‐functional roles of ITCH in cell survival and death

The WW domain‐containing E3 ligase ITCH participates in many signal pathways and contributes to cell survival and death. The first evidence showing the importance of ITCH in human is from the Amish children resulting from the consanguineous marriage. They have a single nucleotide insertion, which causes the frameshift of the coding region and resulting protein truncation. These children have developmental delay and infiltration of immune cells to multiple organs.^53^ Loss of ITCH causes the development of craniofacial abnormalities hepatomegaly, splenomegaly, and inflammation at multiple sites, suggesting the importance of ITCH in the degradation of protein and immune tolerance.

**Figure 2:**
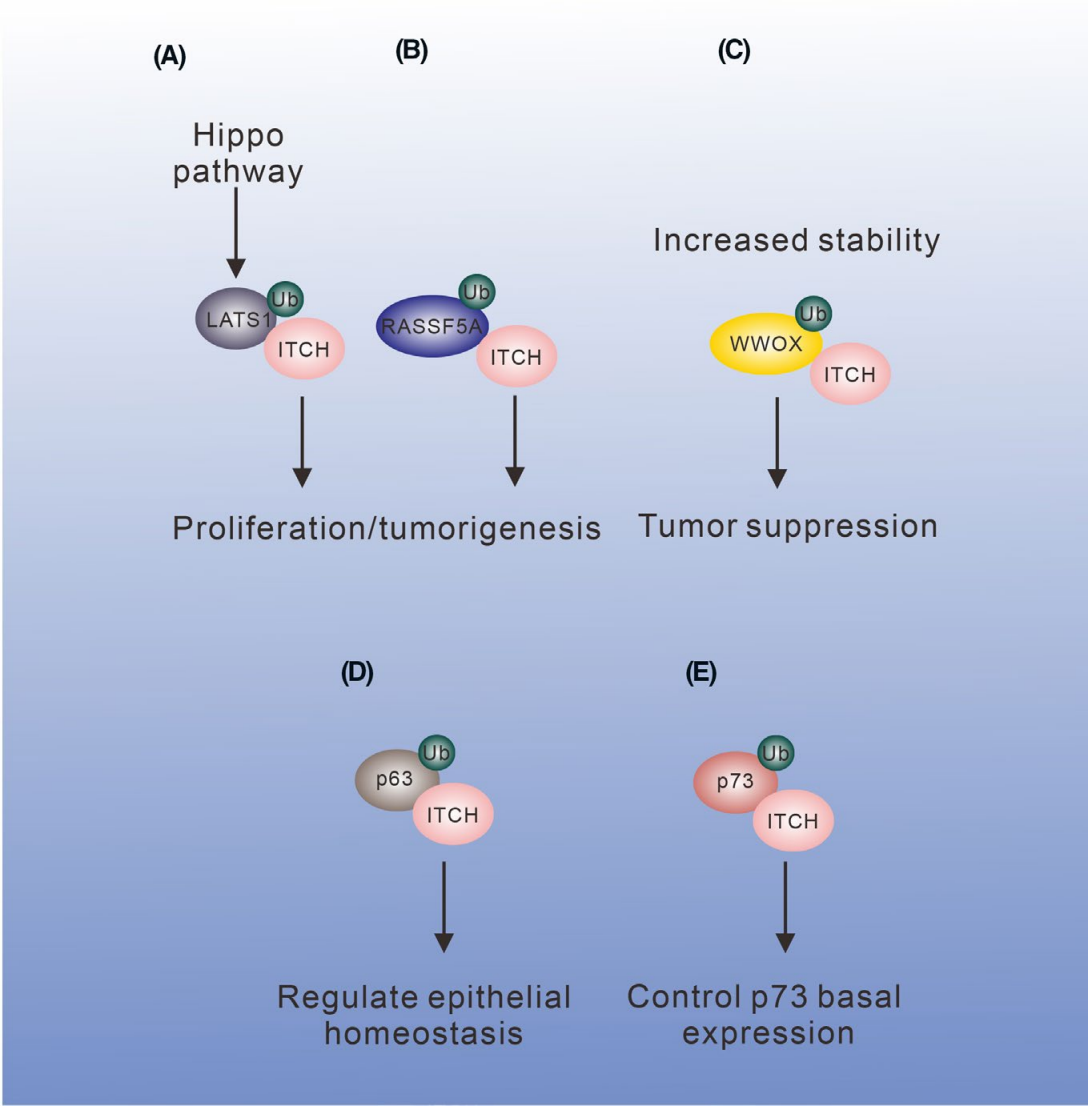
Multi‐functional roles of ITCH in cell survival and death. (A) ITCH participates in the Hippo pathway. ITCH ubiquitinates LATS1 for degradation and promotes tumorigenesis. (B) ITCH increases RASSF5A ubiquitination/degradation and promotes Ras‐induced pro‐apoptotic pathway. (C) ITCH monoubiquitinates WWOX, increases WWOX stability, and enhances its tumor suppressor role. (D, E) ITCH regulates p63 and p73 by ubiquitination for degradation

In the hippo pathway, loss of the tumor suppressor LATS1 is associated with the development of tumors such as soft tissue sarcoma, ovarian cancer, and breast cancer.^54^, ^55^ LATS1 restricts the activity of WW domain‐containing protein YAP/TAZ by phosphorylation and promotes YAP/TAZ degradation to inhibit cell proliferation. ITCH interacts with LATS1 through the PY motifs. This interaction results in the ubiquitination and degradation of LATS1^56^, ^57^, ^58^ (Figure 2A). Loss of LATS1 leads to failure of restricting cell proliferation and contributing to tumorigenesis.

Another signaling involves tumorigenesis is the Ras signaling. An adaptor protein RASS5 is associated with Ras and regulates the downstream signaling. RASSF5A is an isoform of RASS5 and participates in the Ras‐induced pro‐apoptotic pathway. Downregulation of RASSF5A is shown in human cancers.^59^, ^60^ ITCH has been reported to interact with RASSF5A. The WW domains of ITCH bind the PY motif in RASSF5A, thus promoting RASSF5A ubiquitination and degradation (Figure 2B). Interestingly, acetylation of RASSF5A prevents it from being ubiquitinated by ITCH and thus contributes to its stability. Apparently, protein acetylation is crucial in determining whether RASSF5A is subjected to ubiquitination for degradation.^61^

In a proteomic screening, ITCH is shown to be a WWOX binding protein.^62^ The group I sequence (LPxY) in ITCH physically binds the first WW domain in WWOX. However, ITCH does not promote WWOX degradation. ITCH mediates the ubiquitination of WWOX at K63, thereby promoting WWOX stability and its nuclear translocation and apoptotic function^62^ (Figure 2C). On the other hand, WWOX protein level is decreased in the *Itch*‐deficient mouse embryonic fibroblasts (MEFs). The observations suggest that ITCH maintains the stability and anti‐tumor function of WWOX.^62^, ^63^

Phosphorylation of a specific amino acid residue(s) outside the WW domain affects protein function and drives the progression of diseases such as cancer and neurodegeneration. For example, when tumor suppressor WWOX is optimally activated, the resulting pY33‐WWOX plays a homeostatic role in maintaining normal physiology and restricts tumor growth.^64^ During the hyperplasia stage of skin cancer development, pY33‐WWOX levels are increased in vivo.^65^ The raised pY33‐WWOX levels are intended to halt cancer growth. Nuclear accumulation of pY33‐WWOX is facilitated by ITCH^62^ and TRAPPC6A.^66^ As the cells advance further toward cancerous, pY33‐WWOX levels are reduced and pS14‐WWOX increased. Conceivably, ITCH initially binds pY33‐WWOX to stabilize the protein. When pY33 is switched to pS14, ITCH probably dissociates from WWOX. The disappearance of WWOX is often observed when cancer cells are at the metastatic stage. It is likely due to ACK1‐mediated phosphorylation of WWOX at Y287, thus leading to rapid WWOX polyubiquitination and then degradation.^67^

P63 is a substrate for ITCH. Rossi et al (2006) demonstrated that WW domains in ITCH interact with PY motif in p63 and promote p63 ubiquitination and degradation in keratinocytes (Figure 2D). Hence, both ITCH and p63 play important roles in regulating epithelial homeostasis.^68^ ITCH also controls the basal p73 protein level by regulating its ubiquitination/proteasomal degradation (Figure 2E). The interaction requires the third and fourth WW domains of ITCH and the PY motif in p73. However, upon DNA damage response, ITCH is degraded and the resulting p73 accumulation contributes to apoptosis and cell cycle arrest.^69^

Collectively, ITCH participates in numerous pathways, and loss of its expression causes craniofacial abnormalities hepatomegaly and severe inflammation in human; while an increased level of ITCH might otherwise enhance tumorigenesis or enhance the anti‐tumor role of WWOX.

### 3.4 Role of WW domain‐containing E3 ligases in the regulation of p53 protein family

**Figure 3:**
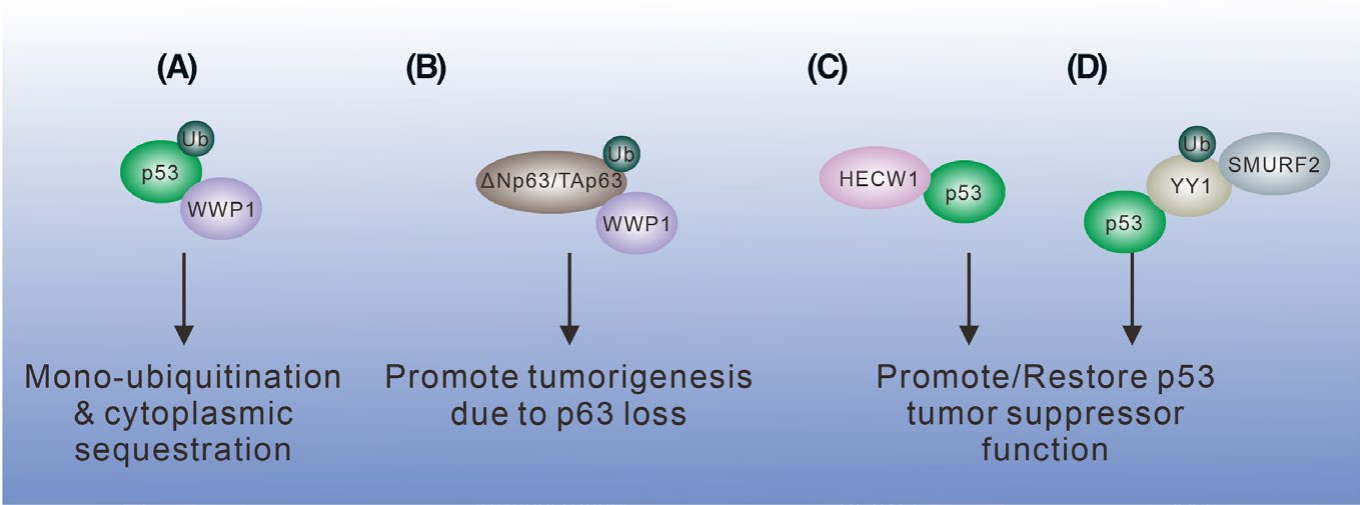
Role of WW domain‐containing E3 ligases in the regulation of the p53 protein family. (A) WWP1 monoubiquitinates p53 and sequesters p53 in the cytoplasm without degradation. (B) WWP1 ubiquitinates both p63 isoforms, and loss of the isoforms causes tumorigenesis. (C) HECW1 does not ubiquitinate p53 but enhances p53‐mediated apoptotic cell death. (D) SMURF2 ubiquitinates YY1 for degradation and releases p53 from the restriction of YY1

Tumor suppressor p53 and family proteins are the binding targets of WWP1. Functionally, WWP1 promotes p53 monoubiquitination and sequestrates p53 protein in the cytoplasm for stabilization (Figure 3A). However, further in‐depth studies are needed to explore the ubiquitination site and molecular mechanism of WWP1/p53‐regulated cell survival or death.^70^

WWP1 binds p63 isoforms ΔNp63α and TAp63α (Figure 3B). Both isoforms originate from the transcription of the alternative promoters. TAp63α promotes cell death, while ΔNp63α has the opposite function. Mutations at the PY motif in both isoforms (Y449F for ΔNp63α and Y543F for TAp63α) abolish the binding interaction with WWP1. Notably, although all the known WW domains (from WW1 to WW4) bind ΔNp63α, the binding between WW1 with ΔNp63α seems to be the strongest among all the WW domains in WWP1. This finding suggests that the WW1 in WWP1 is the major interacting domain with ΔNp63α, and the tandem alignment of the other three WW domains may facilitate the interaction, as evidenced by the fact that the binding of WW1 and WW2 is stronger than that of WW3 and WW4.^71^ Lastly, WWP1 expression negatively correlates with p63 expression in numerous prostate and breast cancer cell lines, suggesting a tumorigenic role of WWP1.

High‐level expression of HECW1 is considered as a favorable prognostic marker for patients with neuroblastoma.^72^ Like other protein members in the NEDD4 family, HECW1 also interacts with p53 (Figure 3C). HECW1 induces cell death by promoting wild‐type p53 activity in neuroblastoma cells.^73^ Neither the HECT domain nor the linker region between the C2 domain and the WW domain of HECW1 is required for the binding with p53. A likely scenario is that this interaction is mediated through the WW domains of HECW1 and the PY motif in p53.^73^

YY1 is reported to be involved in tumorigenesis via inhibiting tumor suppressor p53 function and/or enhancing the tumorigenic role of Ezh2.^74^ SMURF2 binds the PY motif in YY1 for ubiquitination and proteasomal degradation^75^ (Figure 3D). Therefore, SMURF2 helps restore p53 function by rescuing it from the inhibition of YY1.

Together, based on the structural feature of p53, the PY motif is a target for WW domain‐containing proteins. In the case of WW domain‐containing E3 ubiquitin ligases, this group of ubiquitin ligases is involved in the regulation of p53 protein family and control of its stability and function.

### 3.5 Role of WW domain‐containing E3 ligases in the regulation tumor suppressor PTEN

Downregulation or loss of tumor suppressor PTEN has frequently been found in tumor tissues. NEDD4 was shown to downregulate PTEN.^76^ *Pten* gene knockout mice exhibit increased prostatic tumorigenesis. The tumors with reduced PTEN expression inversely correlate with the NEDD4 protein level. Similar to prostate cancer, increased expression of NEDD4 and reduced expression of PTEN have been reported in non‐small‐cell lung carcinomas, further suggesting the role of NEDD4 in promoting PTEN degradation and tumorigenesis.^77^ A recent study reported that the adaptor protein Numb mediates the interaction between NEDD4 and PTEN. Loss of Numb diminishes the binding of NEDD4 with PTEN. That is, the interaction of NEDD4 and PTEN is indirect and requires other proteins.^78^ However, the detailed molecular mechanism regarding how NEDD4 regulates PTEN ubiquitination and degradation and the binding regions in both proteins are largely unknown.

**Figure 4:**
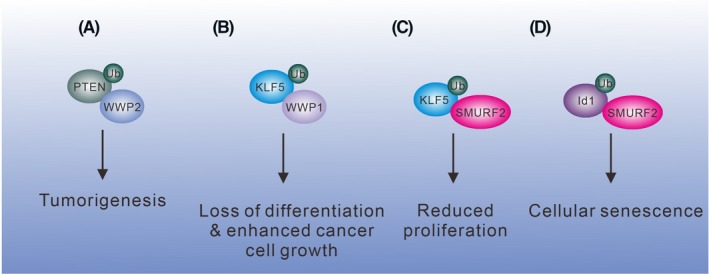
WWP1 and SMURF2 regulate KLF5 protein degradation. (A) WWP2 binds and increases PTEN ubiquitination and degradation for leading to tumorigenesis. (B) KLF5 regulates cell proliferation and differentiation. WWP1 promotes KLF5 degradation and promotes tumorigenesis. (C) SMURF2 ubiquitinates KLF5 and reduces the proliferation of kidney fibroblast‐like cells. (D) Id1 suppresses cellular senescence, and SMURF2 ubiquitinates Id1 and promotes senescence. All the aforementioned ubiquitinated proteins are subjected to degradation

In another report, WWP2 is known as a binding partner to increase PTEN ubiquitination and degradation.^79^ WWP2 binds the PTEN phosphatase domain instead of the conventional PY motif. It is plausible that there is an unidentified protein‐binding module in the phosphatase domain, which shares a similar property with the PY motif. In a *Wwp2* knockout mouse model, convincing evidence revealed that loss of WWP2 expression leads to increased levels of PTEN protein in vivo. Given the potent function of PTEN in tumor suppression, the regulation of PTEN protein level by WWP2 is critical in controlling tumorigenesis^80^ (Figure 4A).

### 3.6 WWP1 and SMURF2 regulate KLF5 protein degradation

The transcription factor KLF5 plays an important role in cell proliferation and differentiation. Loss or deletion of *KLF5* is frequently found in prostate and breast cancers.^81^, ^82^ WWP1 regulates the KLF5 protein level through ubiquitination and proteasomal degradation processes. The WW domains of WWP1 physically bind the PY motif in KLF5 and promote its degradation in cancer cells^83^ (Figure 4B). In addition, WWP1 is frequently amplified in breast, prostate, oral, hepatocellular carcinoma, and gastric carcinoma cells,^84^, ^85^, ^86^, ^87^ suggesting the crucial impact of WWP1 in tumorigenesis.

The other WW domain‐containing E3 ligase SMURF2 also binds the PY motif in KLF5 and mediates KLF5 ubiquitination and degradation. Downregulation of KLF5 decreases its transcriptional activity and reduces cell proliferation in the primary monkey kidney fibroblast‐like cell line^88^ (Figure 4C). Thus, KLF5 plays distinct roles in controlling the proliferation of tumor and normal cells. SMURF2 also promotes the degradation of the transcriptional regulator Id1. Id1 inhibits cellular senescence by repressing p16 expression. SMURF2 ubiquitinates Id1 for proteasomal degradation, thus preventing Id1‐mediated repression of p16 and promoting cellular senescence in human fibroblasts^89^ (Figure 4D).

### 3.7 WWP2 controls the stability of Notch3, EGR2, and TRIF by ubiquitination and degradation

**Figure 5:**
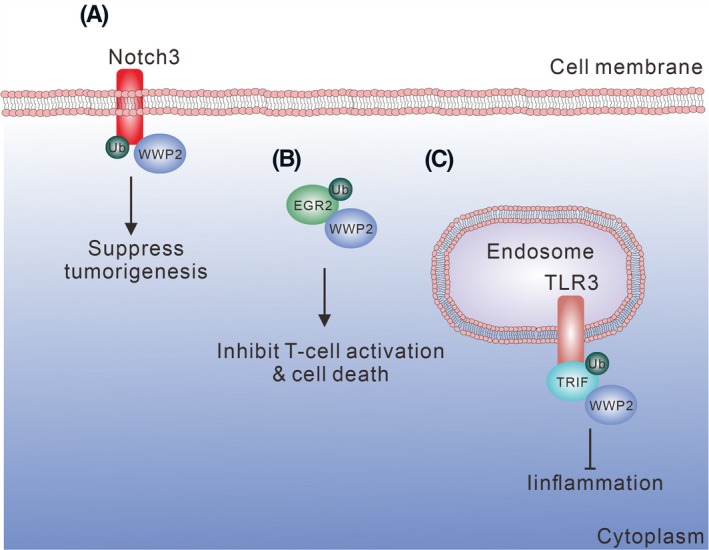
WWP2 regulates the stability of Notch3, EGR2, and TRIF by ubiquitination and degradation. (A) WWP2 ubiquitinates Notch3 and decreases Notch signaling and suppresses tumorigenesis. (B) WWP2 inhibits activation‐induced T cell death by promoting EGR2 degradation. (C) WWP2 inhibits TLR3‐mediated inflammation signaling by promoting TRIF degradation. All the aforementioned ubiquitinated proteins are subjected to degradation

In the Notch signaling, binding of the WW domains in WWP2 with the PY motif in Notch3 leads to ubiquitination and degradation of Notch3 protein (Figure 5A). Ectopically expressed WWP2 decreases Notch3 signaling and results in the cell cycle arrest and reduced tumor volume in an ovarian cancer xenograft model.^90^ WWP2 is also involved in attenuating the activation‐induced death of T cells.^91^ WWP2 binds EGR2 through its WW domains and PY motif in EGR2 to promote EGR2 ubiquitination and degradation, thereby reducing EGR2‐induced Fas ligand expression after T cell activation (Figure 5B). In the innate immunity, the WW domains of WWP2 interact with the *N*‐terminal region and TIR domain of TRIF (Figure 5C). This interaction promotes TRIF K48‐linked ubiquitination and degradation upon TLR3 activation. Therefore, WWP2 inhibits the TLR3‐mediated NF‐κB and IRF‐3 activation. Furthermore, *Wwp2* gene knockout mice have an increased innate immune response and are susceptible to poly(I:C)‐induced death.^92^

### 3.8 Ubiquitination site switching

Switching at the ubiquitination site regulates protein activity. One example is that the switch of ubiquitination site at K63 to K48 in TAK1 terminates the TAK1 activity. ITCH and Cyld regulate this switch. The WW domains of ITCH bind the PY motif in Cyld.^93^ Both proteins form a complex and promote the switch of K63 to K48 ubiquitination on TAK1. This switch terminates the TAK1‐mediated inflammatory response in bone marrow‐derived macrophages.^93^ Mice deficient in *Itch* or *Cyld* gene develops inflammation in numerous tissues such as in lung and spleen.^93^ Mice deficient in *Cyld* is susceptible to chemically induced tumorigenesis. In addition, both *Itch‐* and *Cyld*‐deficient mice have a higher frequency in developing lung cancer metastasis. Thus, ITCH‐ and Cyld‐controlled ubiquitination are crucial for the regulation of inflammatory response and tumorigenesis.^93^

## 4 ROLE OF WW DOMAIN‐CONTAINING E3 LIGASES IN NEUROLOGICAL/RARE DISEASES AND OTHER CELLULAR FUNCTION

Deficiency of a specific WW domain‐containing protein or alterations in the WW domains affects the binding with ligands. The consequence of aberrant signaling may link to the generation of neurological disorders or other diseases. For example, WWOX deficiency is known to cause severe neural disease, including ataxia, severe early‐onset epileptic encephalopathy, global developmental delay, acquired microcephaly, epilepsy, and early death.^62^, ^64^

### 4.1 Role of NEDD4 in membrane protein homeostasis and neurodegeneration

**Figure 6:**
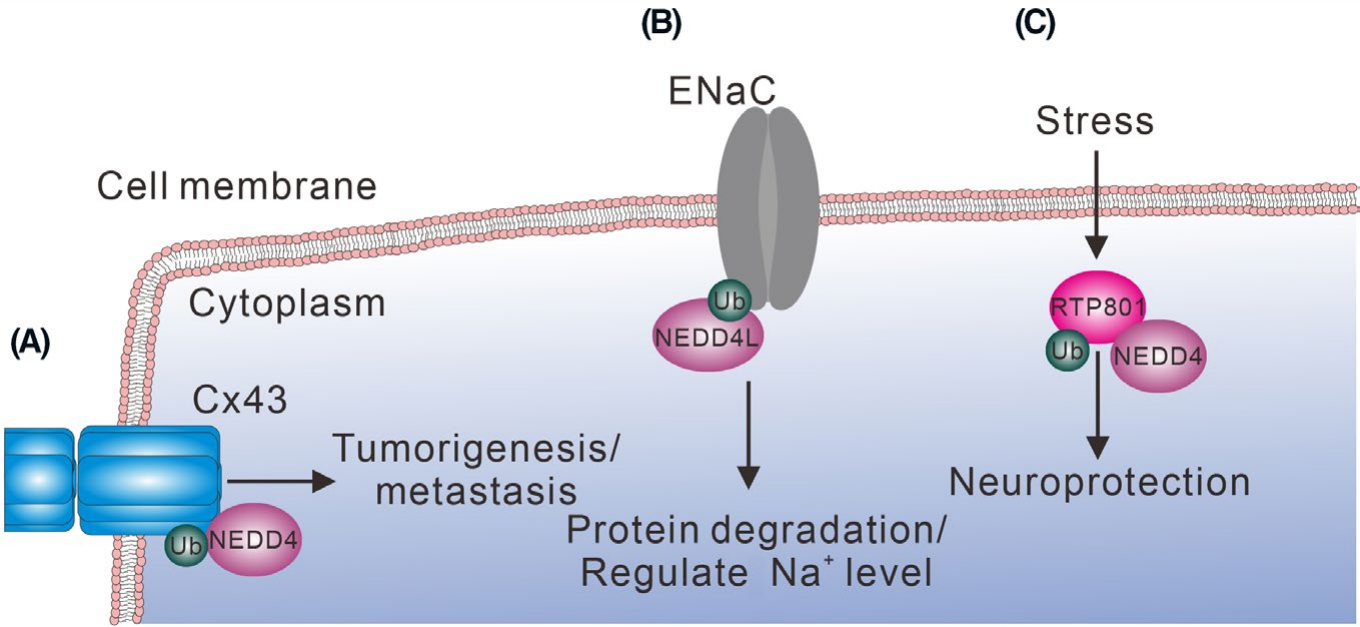
Role of NEDD4 in the membrane protein homeostasis and neurodegeneration. (A) NEDD4 mediates Cx43 degradation, and loss of Cx43 promotes tumorigenesis. (B) NEDD4L ubiquitinates and mediates ENaC degradation. (C) NEDD4 ubiquitinates the pro‐apoptotic protein RTP801 and prevents it from causing neuronal cell death

Connexin 43 (Cx43) is one of the components of gap junction. Loss of Cx43 causes neonatal lethality owing to the defects in the heart and nervous systems during development.^94^, ^95^, ^96^ Also, mutation and loss of Cx43 expression promote tumorigenesis and metastasis in the DMBA‐induced tumor model.^97^ NEDD4 is the first ubiquitin ligase that has been reported to mediate internalization and degradation of Cx43^98^ (Figure 6A). Their interaction is mediated through the first, second, and third WW domains in NEDD4, and the PY motif is located at the *C*‐terminus of Cx43. MAPK signaling cascade induces phosphorylation of the serine residues at amino acids 279 and 282 in Cx43, which significantly increases the binding affinity of the tandemly aligned WW domains to the phosphor‐PY motif (similar to group IV WW domain substrate). Indeed, the second WW domain of NEDD4 acquires a significantly enhanced binding to the phospho‐PY motif.^98^, ^99^ This change in the binding affinity affects the NEDD4‐regulated protein turnover of gap junction component Cx43. NEDD4 deficiency leads to Cx43 accumulation in the plasma membrane. While increased gap junction formation is associated with neuroinflammation and neuronal cell death, functional blockade of Cx43 promotes neuron cell survival and reduces inflammation.^100^

In addition to gap junctions, NEDD4 also regulates the amiloride‐sensitive epithelial sodium channel (ENaC) protein level and its activity. Mutant ENaC, lacking the *C*‐terminal PY motif or containing a disrupted consensus PY motif due to missense mutations, possesses an increased sodium channel activity and membrane retention that results in hypertension in Liddle syndrome.^101^, ^102^, ^103^, ^104^ Similar to the structure of NEDD4, the other WW domain‐containing E3 ligase, NEDD4L, catalyzes ubiquitination and promotes cell surface ENaC degradation (Figure 6B),^105^ suggesting that both NEDD4 and NEDD4L have the similar role in regulating ENaC turnover.

In Parkinson's disease (PD), reduced expression of NEDD4 in the nigral neurons from the PD brains has been documented.^104^ NEDD4 mediates the ubiquitination and degradation of the pro‐apoptotic protein RTP801 (Figure 6C). RTP801 possesses a proline‐rich sequence near its *N*‐terminus and is significantly upregulated in the brain of PD patients. Loss of NEDD4 increases the susceptibility of neuronal cells to RTP801‐induced cytotoxicity.^106^

### 4.2 Regulation of membrane voltage‐gated sodium channels (Na_v_) by NEDD4L

Voltage‐gated sodium channels (Na_v_) are transmembrane proteins that regulate the flow of sodium ions and control the initiation of the action potential. These channels are abundant in nerve and muscle cells, in which ion flow and action potential are critical for sustaining cellular function. From sequence analysis, there are 7 Na_v_ subunits among the 10 Na_v_ having the PY motif at the *C*‐terminus.^107^, ^108^ One of the subunit Na_v_1.5 has been studied extensively. NEDD4L interacts with the *C*‐terminal PY motif and promotes the ubiquitination and degradation of Na_v_1.5 in HEK293 cells.^107^, ^108^

In addition to Na_v_1.5, Na_v_1.7 and Na_v_1.8 are involved in neuronal hyperexcitability and sensation of pain. Genetic alteration of Na_v_1.7 and Na_v_1.8 contributes to the development of neuropathic pain owing to the enhanced function of both sodium channels.^109^, ^110^ NEDD4L has been identified as a regulator of both Na_v_1.7 and Na_v_1.8 and negatively regulates the levels of both sodium channels in dorsal root ganglia (DRG). Interestingly, NEDD4L expression is downregulated after nerve injury and results in the accumulation of Na_v_1.7 and Na_v_1.8 in DRG, where enhanced sodium influx causes neuron excitability and hypersensitivity to pain.^111^, ^112^

In conclusion, both NEDD4 and NEDD4L play an important role in regulating the stability of membrane proteins, which mainly regulate the transportation of molecules between cell‐cell and ions from extracellular space to the cytosol. Deficiency in NEDD4 or NEDD4L contributes to the accumulation of ion channels in the membrane, and the enhanced ion flow causes neuronal diseases.

### 4.3 Role of WWP1 in the development of Huntington's disease

WWP1 regulates p53 family proteins such as p53 and p63. WWP1 expression inversely correlates with the p53 and p63 protein levels. A recent study reported that WWP1 ubiquitinates huntingtin (Htt), and that mutation and aggregation of Htt are associated with Huntington's disease (HD) progression.^113^ WWP1 ubiquitinates the mutant Htt through K63 ubiquitination but does not promote its degradation. Instead, WWP1 aggregates with mutant Htt and exacerbates the disease progression.^113^ Although other E3 ubiquitin ligases might also be involved in the clearance of mutant Htt, in that case, interference of interaction between WWP1 and mutant Htt may reduce protein aggregation and disease progression.

### 4.4 HECW1‐mutant SOD1‐TRAPδ complex in processing mutant SOD1

HECW1 was first isolated from human neuroblastoma tissues, and this protein is abundant in neuronal tissues such as brain and spinal cord.^72^ The mutant SOD1 and TRAP‐δ are the binding partners of HECW1. HECW1 physically interacts with TRAP‐δ through its two WW domains. However, HECW1 binds mutant SOD1 through a linker region between C2 and WW domains. Although this HECW1‐mutant SOD1‐TRAPδ complex promotes degradation of mutant SOD1, these proteins tend to form the neuronal Lewy body‐like hyaline inclusion, which is a hallmark of the disease severity of familial amyotrophic lateral sclerosis.^72^, ^114^ Indeed, certain proteins that are essential for normal cellular functions are sequestrated in the protein aggregates. For example, Dvl1 is found in the protein inclusion, and this sequestration impairs the signaling of both Wnt/β‐catenin and JNK/c‐Jun pathways and causes cytotoxicity.

### 4.5 HECW2 binds p73 and AMOTL1

**Figure 7:**
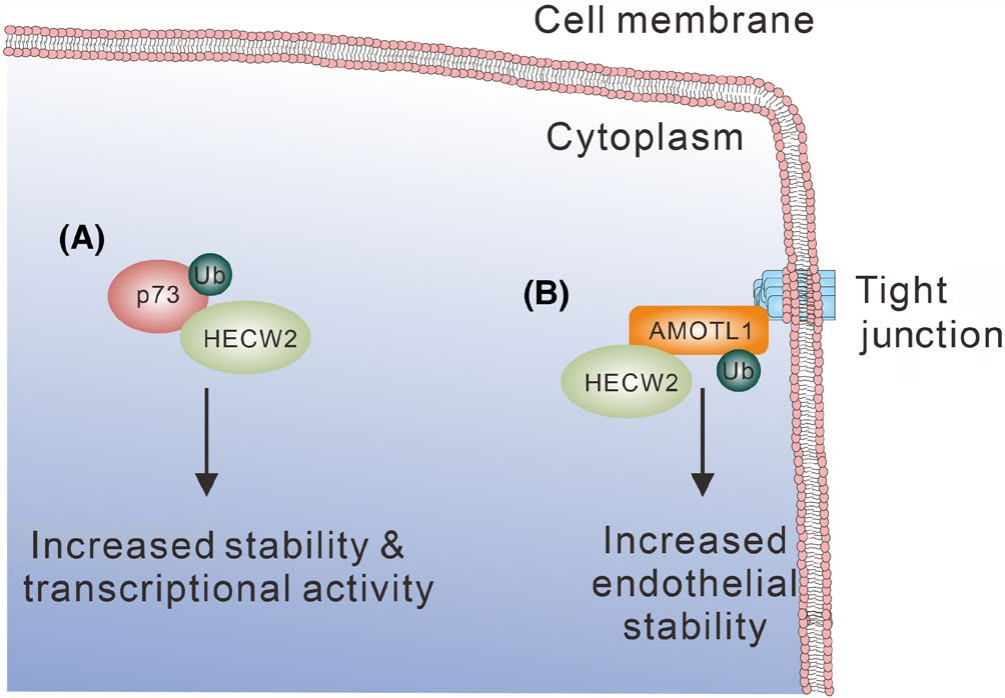
Role of HECW2 in regulating p73 and AMOTL1 stability. (A) HECW2 ubiquitinates p73 and increases p73 stability and transcriptional activity. (B) HECW2 ubiquitinates AMOTL1 and promotes its stability in endothelial cells

HECW2 specifically binds p73 but not p53.^115^ The WW domains of HECW2 recognize the *C*‐terminal PY motifs of p73, and this interaction promotes p73 ubiquitination (Figure 7A). Instead of degradation, p73 ubiquitination by HECW2 increases its stability and transcriptional activity.^115^ AMOTL1, a component of tight junctions, regulates endothelial cell junctional structure, permeability, polarity, migration, capillary formation, and vascular stability. Similarly to the regulation of p73 activity, binding of HECW2 with AMOTL1 promotes AMOTL1 K63‐ubiquitination and increases stability in endothelial cells, while the loss of AMOTL1 promotes angiogenic sprouting^116^ (Figure 7B).

Loss of HECW2 decreases the activation of GDNF/Ret downstream Akt signaling in human Hirschsprung's disease.^117^, ^118^ The GDNF/Ret signaling is important for kidney development.^119^ Loss of HECW2 causes neonatal death of mice within two weeks after birth. These mice showed a decreased GDNF/Ret signaling, low body weight, and defects in intestinal and kidney development.^120^ Although the molecular mechanism remains unclear, additional protein modifications such as neddylation may be involved in regulating HECW2 activity.

### 4.6 SHH signaling and SMURF1/2 in neural development

**Figure 8:**
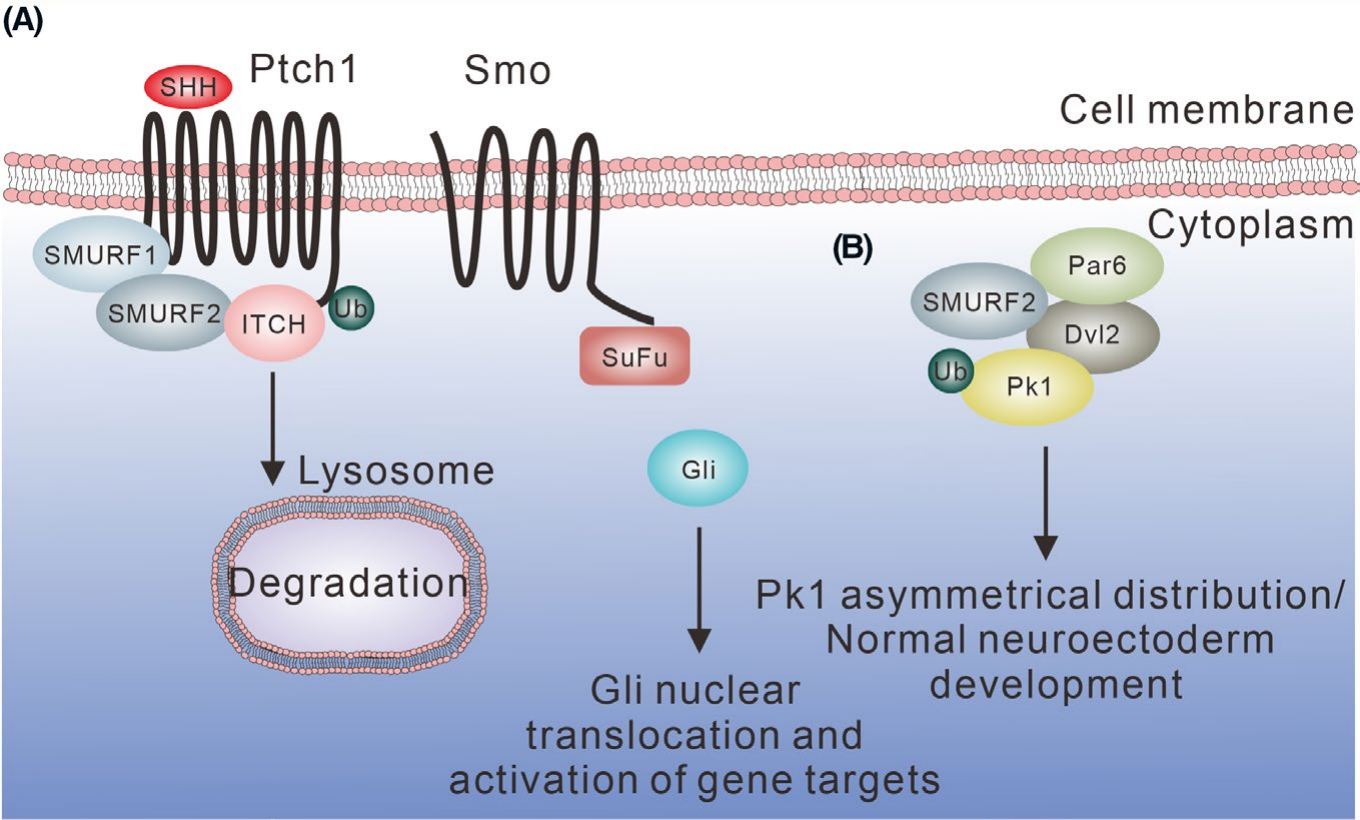
SHH signaling and SMURF1/2 in neuronal development. (A) SMURF1 and SMURF2 ubiquitinate Ptch1 for degradation and release Smo and Gli, thereby increasing the expression of Gli‐targeted genes. (B) SMURF2 promotes the ubiquitination of Pk1 for degradation, thus leading to the asymmetrical distribution of Pk1 during normal development

Sonic Hedgehog signaling is important for brain development, whereas an increased activity of SHH signaling induces tumorigenesis.^121^ The SHH receptor Patched (Ptch1) restrains smoothened (Smo) from releasing the Gli zinc‐finger transcription factors and inhibits SHH signaling. Upon the binding of Ptch1 with its ligand SHH, Gli is released and translocates to the nucleus to drive gene expression. SMURF1 and SMURF2 participate in SHH signaling.^122^ Both SMURF1 and SMURF2 bind the PY motif of Ptch1 and ubiquitinate Ptch1 for internalization and proteasomal degradation (Figure 8A). ITCH also regulates SHH signaling via binding to the PY motifs in the intracellular *C*‐terminal domain and the central loop region of Ptch1 with its WW domains. ITCH promotes Ptch1 ubiquitination and degradation, thereby modulating the apoptotic function of Ptch1.^123^ ITCH‐mediated downregulation of Ptch1 allows the release of Gli from inhibition and drives the expression of Gli‐target genes necessary for normal brain development during the embryonic stage. Loss of SMURF1/2 abolishes cerebellum development. In addition, mice lacking both SUMRF1 and SMURF2 exhibit impaired cell proliferation during cerebellar organogenesis and show early embryonic lethal, again suggesting an essential role of SMURF1/2 in brain development.^122^

Moreover, SMURFs participate in the noncanonical Wnt signaling and are one of the components of the PCP complex. Upon stimulation of noncanonical Wnt ligand Wnt5A, SMURF2 forms a tripartite complex with phosphorylated Dvl2 and Par6. The PY motif in Dvl2 is required for the binding with WW domains in SMURF2. This SMURF2‐phospho‐Dvl2‐Par6 interacts with Pk1 and promotes Pk1 ubiquitination and degradation (Figure 8B). These results in the asymmetrical distribution of Pk1 and is required for the normal development of neuroectoderm.^124^

## 5 RELATIONSHIPS BETWEEN CANCER AND NEURODEGENERATION

There is an inverse correlation between the progression of cancers and neurodegenerative diseases.^125^, ^126^, ^127^ Cancer patients may have a reduced risk of developing neurodegenerative diseases such as dementia, Alzheimer's disease, Parkinson's disease, and Huntington's disease in general. Supporting evidence reveals that tumor suppressors p53 and PTEN might have a role in the disease progression.^128^, ^129^, ^130^, ^131^ Albeit loss of PTEN expression contributes to cancer progression, inhibition of PTEN rescues the normal synaptic function and cognition in animal model.^128^ As a gatekeeper, p53 controls cell cycle progression, DNA damage response, and tissue development. However, elevated p53 protein level in the brain of patients with Parkinson's disease and Huntington's disease cause neuronal cell death.^129^, ^130^ Target deletion or disruption of p53 function suppresses neurodegeneration and neurobehavioral abnormalities in the animal model.^129^ Particularly, both PTEN and p53 have positive roles in tumor suppression and neurodegeneration and are in contrast to the aforementioned findings that cancer patients have a reduced risk of developing neurodegenerative diseases.^132^, ^133^ In the notion of Parkinson's disease, there is an increased risk of developing melanoma, breast tumor, uterine, and renal cancer.^127^

Based on the biochemical evidence, PTEN and p53 interact with NEDD4, WWP1, WWP2, and HECW1, and the binding has been shown to be involved in neuronal cell death and diseases.^72^, ^106^, ^113^, ^114^ These findings suggest that alterations of WW domain‐containing E3 ligases in binding and signaling may contribute to the development of cancers and neurodegenerative disease. Intriguingly, the involved pathways in the WW domain‐containing E3 ligases enrich with EGFR, TGF‐β, and BMP signaling,^23^, ^25^, ^31^, ^34^, ^39^, ^40^, ^41^, ^47^ implying that the ubiquitin ligases, including WW domain‐containing E3 ligases, might play a role as a double‐edged sword in tumorigenesis and the development of neuronal diseases.

## 6 CONCLUDING REMARKS

**Figure 9:**
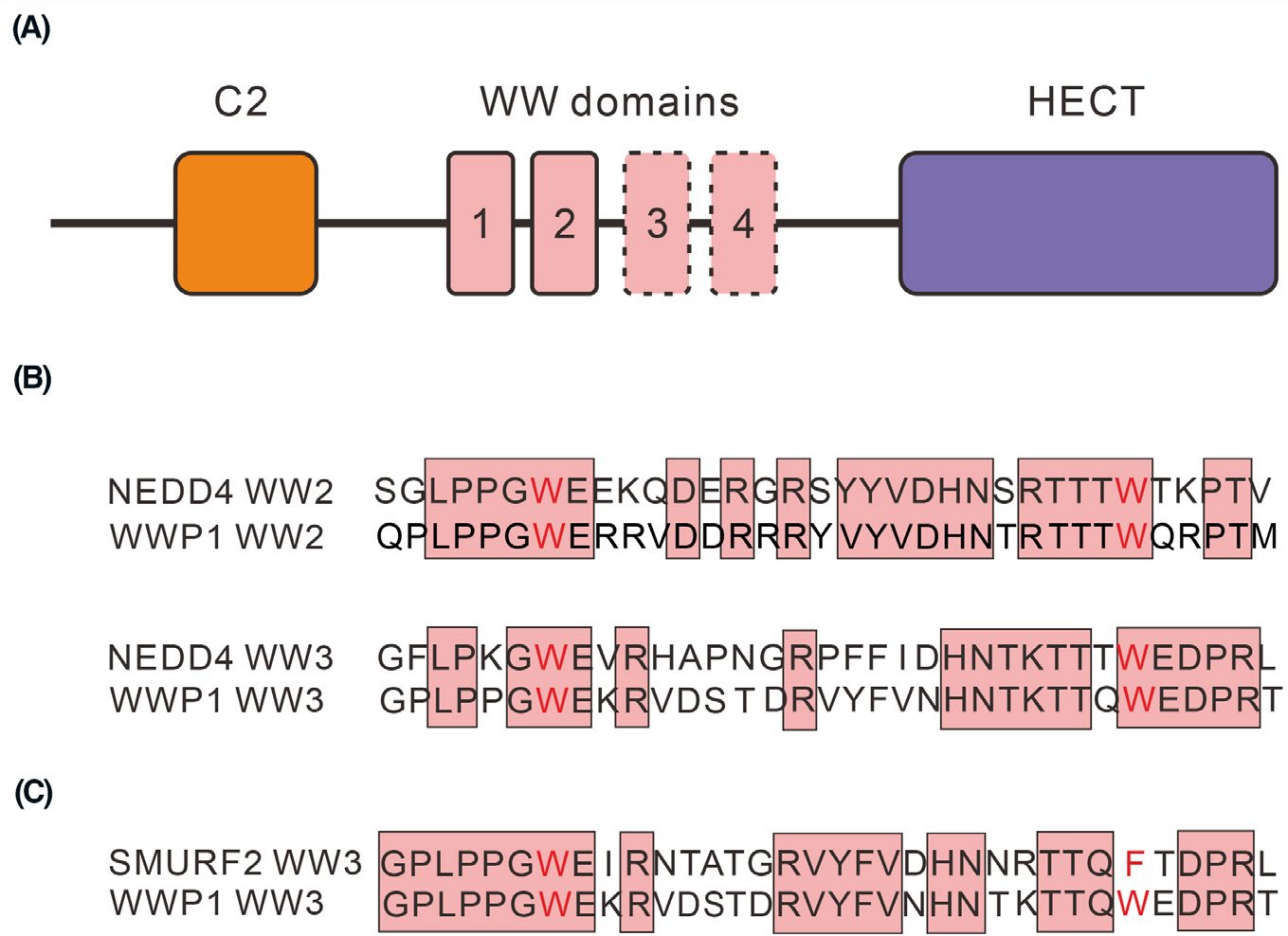
Structure of WW domain‐containing E3 ligases. (A) WW domain‐containing E3 ligases share a common structure, an *N*‐terminal C2 domain, and a *C*‐terminal HECT domain, which catalyzes the transfer of ubiquitin molecule. The WW domains locate between the C2 domain and the HECT domain. There are two to four WW domains in this central region among the nine WW domain‐containing E3 ligases, annotated as WW1, WW2, WW3, and WW4. The WW3 and WW4 are presented in some WW domain‐containing E3 ligases, as indicated by the dashed line. The WW domains in this region play an important role in recognizing its binding proteins. (B) Sequence alignment of WW domains in SMURF2 and WWP1. The WW2 and WW3 in SMURF2 and WWP1 share the high sequence similarity and have the same binding protein ErbB4/HER4. (C) Sequence alignment of WW domains in SMURF2 and WWP1. The WW3 in both proteins shares high sequence similarity and has the same binding protein Smad7

WW domains are highly conserved protein‐binding modules that interact with a distinct subset of proteins possessing proline‐rich sequences (Table 1). Cumulative biochemical evidence suggests that different groups of WW domains preferentially bind a distinct proline‐rich sequence (Figure 9A, Tables 1 and 2). This property strengthens their potential involvement in signal pathways. For example, the WW domains in NEDD4 and WWP1 share a high similarity (Figure 9B), and the biochemical evidence shows that NEDD4 and WWP1 co‐regulate ErbB4/HER4.^21^, ^22^, ^31^ Also, similar WW domains share a common ligand. For example, WW domains in SMURF2 and WWP1 share a common ligand Smad7 (Figure 9C).^134^, ^135^ WW domain‐containing protein YAP1 also bears the WW domain structure highly similar to that of SMURF2, and both have a common ligand Smad7.^136^ Taken together, proteins with strong sequence homologies in the WW domains tend to participate in the overlapping signaling pathways. In order to prevent cross‐talk that causes chaos, fine‐tune regulation of the levels of individual proteins in different tissues and development stages seems to be a feasible way to streamline the signaling paths.

**Table 2:** Sequences of WW domains in WWOX and WW domain‐containing E3 ligases

Name	WW1	WW2	WW3	WW4
NEDD4	SPLPPGWEERQDILGRTYYVNHESRRTQWKRPTP	SGLPPGWEEKQDERGRSYYVDHNSRTTTWTKPTV	GFLPKGWEVRHAPNGRPFFIDHNTKTTTWEDPRL	GPLPPGWEERTHTDGRIFYINHNIKRTQWEDPRL
NEDD4L	PPLPPGWEEKVDNLGRTYYVNHNNRTTQWHRPSL	PGLPSGWEERKDAKGRTYYVNHNNRTTTWTRPIM	SFLPPGWEMRIAPNGRPFFIDHNTKTTTWEDPRL	GPLPPGWEERIHLDGRTFYIDHNSKITQWEDPRL
ITCH	GPLPPGWEKRTDSNGRVYFVNHNTRITQWEDPRS	KPLPEGWEMRFTVDGIPYFVDHNRRTTTYIDPRT		
WWOX	DELPPGWEERTTKDGWVYYANHTEEKTQWEHPKT	GDLPYGWEQETDENGQVFFVDHINKRTTYLDPRL		
WWP1	ETLPSGWEQRKDPHGRTYYVDHNTRTTTWERPQP	QPLPPGWERRVDDRRRVYYVDHNTRTTTWQRPTM	GPLPPGWEKRVDSTDRVYFVNHNTKTTQWEDPRT	EPLPEGWEIRYTREGVRYFVDHNTRTTTFKDPRN
WWP2	DALPAGWEQRELPNGRVYYVDHNTKTTTWERPLP	RPLPPGWEKRTDPRGRFYYVDHNTRTTTWQRPTA	PLPPGWEKRQDNGRVYYVNHNTRTTQWEDPRT	PALPPGWEMKYTSEGVRYFVDHNTRTTTFKDPRP
HECK1	EPLPPNWEARIDSHGRVFYVDHVNRTTTWQRPTA	LELPRGWEIKTDQQGKSFFVDHNSRATTFIDPRI		
HECK2	EALPPNWEARIDSHGRIFYVDHVNRTTTWQRPTA	LELPRGWEMKHDHQGKAFFVDHNSRTTTFIDPRL		
SMURF1	PELPEGYEQRTTVQGQVYFLHTQTGVSTWHDPRI	GPLPPGWEVRSTVSGRIYFVDHNNRTTQFTDPRL		
SMURF2	NDLPDGWEERRTASGRIQYLNHITRTTQWERPTR	PDLPEGYEQRTTQQGQVYFLHTQTGVSTWHDPRV	GPLPPGWEIRNTATGRVYFVDHNNRTTQFTDPRL	

WW domain‐containing proteins participate in many biological and cellular processes. Gene amplification, deletion, and mutation of WW domain‐containing proteins are frequently found in human cancers and neuronal diseases, again suggesting their important roles in disease development (Table 1). The E3 ligases are thought to be undruggable targets owing to their high involvement in the cellular process and protein homeostasis. Nowadays, with the advance in protein structural studies, many protein‐protein interaction analyses and computational prediction approaches have narrowed down the region that are required for the functional motifs in the protein‐protein binding. In addition, the first proteasome inhibitor Bortezomib has shown its anti‐tumor potency and been approved for treating myeloma and lymphoma.^137^, ^138^ It sheds an unprecedented light on the concept that targeting UPS is possible. To be more specifically minimize the side effects, targeting to a particular signaling or protein molecule is envisaged. Although it was not found in cancer, blocking the binding of NEDD4 WW domains with viral PPxY budding domain prevents virus egress.^139^ The development of this small molecule drug against viruses such as Ebola and Marburg is prospective.

Intriguingly, WW domain‐containing E3 ligases participate in many signal pathways in tumorigenesis and cell death. Selective inhibition of WW domain‐containing E3 ligase has been a novel strategy in the field of cancer therapy. For example, WWP1 has been found amplified in numerous tumors, while downregulation of WWP1 attenuates leukemic cell growth.^140^ Similar to WWP1, a small‐molecule inhibitor of WWP2 has been investigated and might have an anti‐tumor function.^141^ Furthermore, enhancing or disruption of the protein‐protein interactions between WW domain‐containing E3 and its substrate is a novel strategy to treat cancers and diseases.

## CONFLICT OF INTEREST

The authors declare no conflict in interest associated with the article.

## AUTHOR CONTRIBUTIONS

SSH wrote the article, LJH revised the article, and NSC polished the scientific writing and made the final revision. All authors approved the article prior to submission.

## COMPLIANCE WITH ETHICAL STANDARDS

This review article does not involve in using animal or human subjects.
